# Biomaterials and Tissue Engineering Strategies for Mastoid Obliteration: A Systematic Review of the Clinical and Preclinical Evidence

**DOI:** 10.3390/jcm15186952

**Published:** 2026-09-08

**Authors:** Giovanni Freda, Carla Laria, Antonietta Mallardo, Gennaro Auletta, Alessandra Murri, Vito Pontillo, Serena Danti, Nicola Serra, Andrea De Vito, Nicola Antonio Adolfo Quaranta, Stefano Berrettini, Anna Rita Fetoni

**Affiliations:** 1 Audiology Section, Department of Neuroscience and Reproductive Sciences and Dentistry, University of Naples Federico II, 80131 Naples, Italy; dott.giovannifreda@gmail.com (G.F.); carla.laria@unina.it (C.L.); toniam951@gmail.com (A.M.); auletta@unina.it (G.A.); annarita.fetoni@unina.it (A.R.F.); 2 Otolaryngology Unit, Department of Translational Biomedicine and Neurosciences (DiBraiN), University “Aldo Moro” of Bari, 70124 Bari, Italy; alessandra.murri@uniba.it (A.M.); pontillovito@gmail.com (V.P.); nicolaantonioadolfo.quaranta@uniba.it (N.A.A.Q.); 3 Department of Civil and Industrial Engineering, University of Pisa, 56126 Pisa, Italy; serena.danti@unipi.it; 4 Department of Surgical, Medical, Molecular Pathology and Critical Area, University of Pisa, 56126 Pisa, Italy; andrea.devito.orl@gmail.com (A.D.V.); stefano.berrettini@unipi.it (S.B.); 5 Unit of Otolaryngology, Audiology and Phoniatrics, Azienda Ospedaliero-Universitaria Pisana, 56124 Pisa, Italy; 6 Hearing and Balance Unit, Department of Head and Neck, Federico II University Hospital, 80131 Naples, Italy

**Keywords:** mastoid obliteration, biomaterials, S53P4 bioactive glass, tissue engineering, bone regeneration, cholesteatoma

## Abstract

**Background/Objectives:** Mastoid obliteration is being increasingly used to reduce cavity-related morbidity after mastoidectomy. Alongside autologous grafts, synthetic biomaterials and tissue-engineering approaches have emerged as potential alternatives. This systematic review synthesized the clinical and preclinical evidence on biomaterials for mastoid obliteration, focusing on efficacy, safety, biological integration, and translational potential. **Methods:** A Preferred Reporting Items for Systematic Reviews and Meta-Analyses (PRISMA)-compliant search of PubMed, Scopus, and Web of Science identified studies published between January 2010 and May 2026. Eligible studies evaluated biomaterials for mastoid obliteration in clinical settings or experimental models. Data on study design, biomaterial type, intervention, outcomes, complications, and biological endpoints were extracted. Risk of bias was assessed using the Risk of Bias in Non-randomized Studies of Interventions (ROBINS-I), Revised Cochrane Risk of Bias Tool for Randomized Trials (RoB 2), and the Systematic Review Centre for Laboratory Animal Experimentation (SYRCLE) risk-of-bias tool. **Results:** Twenty-eight studies were included (19 clinical, 9 preclinical). Clinical evidence supported mastoid obliteration as an effective strategy for improving cavity stability, reducing chronic otorrhea, promoting epithelialization, and aiding cholesteatoma control. Outcomes appeared to depend more on successful cavity elimination than on the superiority of a specific biomaterial. Autologous materials showed reliable and reproducible results, while S53P4 bioactive glass demonstrated comparable effectiveness with additional antibacterial properties. Hydroxyapatite-based materials and allografts showed more variable outcomes. Biologically enhanced approaches, including concentrated growth factors and composite constructs, yielded promising but limited clinical evidence. Preclinical studies indicated that conventional biomaterials are primarily osteoconductive, whereas composite scaffolds, bone morphogenetic protein-2 (BMP-2) delivery systems, and stem cell-based strategies may enhance osteogenesis and tissue integration. However, clinical translation remains limited. **Conclusions:** Mastoid obliteration is an effective reconstructive strategy whose success depends mainly on stable cavity reduction and adequate epithelial coverage. Autologous materials and S53P4 bioactive glass currently have the strongest clinical support, whereas advanced tissue-engineering approaches require further validation through prospective comparative studies, standardized outcomes, long-term follow-up, and translational research.

## 1. Introduction

Mastoid obliteration has become an established surgical strategy to mitigate the long-term morbidity associated with canal wall down (CWD) mastoidectomy. By reducing the volume of the exteriorized mastoid bowl and restoring a more stable, self-cleaning anatomy, obliteration aims to limit chronic otorrhea, debris retention, recurrent infection, caloric-induced vertigo, water intolerance, and the need for repeated outpatient debridement or lifelong cavity care [1,2]. These clinical benefits have made mastoid obliteration an increasingly relevant adjunct in the surgical management of chronically diseased ears, particularly in patients at risk of unstable or troublesome postoperative cavities.

Historically, mastoid reconstruction has relied on autologous or local materials, including bone pâté, bone dust, cartilage, periosteal or musculoperiosteal flaps, and soft tissue grafts. These materials are readily available, biocompatible, and clinically familiar; however, they were originally conceived mainly as volume-replacing fillers rather than biologically active regenerative constructs. Their long-term performance may therefore be influenced by material resorption, variable structural stability, incomplete epithelialization, persistent inflammatory or infected spaces, and the ongoing challenge of detecting residual or recurrent cholesteatoma in an obliterated cavity [3,4].

In parallel, advances in biomaterials science and tissue engineering have introduced a new conceptual framework for mastoid reconstruction, moving from passive cavity filling toward biologically guided tissue regeneration. In this context, an ideal scaffold should not only provide mechanical support and spatial stability, but also promote cellular adhesion, vascular ingrowth, controlled degradation, and progressive integration with host bone [5,6]. Bioactive glass, calcium orthophosphates such as hydroxyapatite and tricalcium phosphate, and composite polymer-based scaffolds have therefore attracted growing interest because of their osteoconductive properties, surface reactivity, and potential to support bone ingrowth. Moreover, biologically enhanced approaches incorporating growth factors, blood-derived products, or mesenchymal stem/stromal cells may further promote osteogenesis and tissue integration, suggesting a shift toward more regenerative strategies in otologic reconstruction [7,8,9].

Despite these developments, the available evidence remains fragmented and methodologically heterogeneous. Clinical studies provide essential information on safety, cavity stability, infection control, epithelialization, recurrence, and patient-related outcomes, but they often differ substantially in surgical technique, material formulation, follow-up duration, and outcome definitions. Conversely, preclinical investigations offer mechanistic insight into biocompatibility, osteoconduction, osteoinduction, antibacterial behavior, scaffold architecture, and bone regeneration, but their translational relevance to the complex anatomy and disease environment of the human mastoid remains uncertain [10,11]. As a result, clinical and experimental evidence are often considered separately, limiting a comprehensive understanding of how biomaterial properties translate into surgical performance.

Therefore, an integrated synthesis of both clinical and preclinical evidence is needed to define the current role of biomaterial-based mastoid obliteration, distinguish structural filling from true biological reconstruction, and identify which material characteristics may meaningfully influence clinical outcomes and future translational development.

The objective of this systematic review was to critically synthesize the current clinical and preclinical evidence on biomaterials and tissue engineering strategies for mastoid obliteration after mastoidectomy. Specifically, this review aimed to evaluate and compare autologous, allogeneic, and synthetic materials in terms of clinical effectiveness, safety, cavity stability, infection control, cholesteatoma recurrence, epithelialization, and material-related complications. In parallel, it sought to analyze preclinical evidence on biocompatibility, osteoconduction, osteoinduction, antibacterial activity, scaffold architecture, growth factor delivery, and cell-based regeneration. By integrating surgical outcomes with mechanistic and translational data, this review aimed to clarify the current level of evidence, identify methodological limitations, and outline future research priorities for biologically informed mastoid reconstruction.

## 2. Materials and Methods

### 2.1. Study Design

This systematic review was conducted in accordance with the Preferred Reporting Items for Systematic Reviews and Meta-Analyses (PRISMA) guidelines [12]. The aim was to identify and critically synthesize clinical and preclinical evidence on biomaterials used for mastoid obliteration, with particular emphasis on clinical performance, safety, biological integration, and translational relevance. Special attention was given to synthetic and bioactive biomaterials, including bioactive glass, because of their increasing use as alternatives to conventional autologous grafting techniques in otologic reconstruction [13,14]. Both clinical studies and preclinical investigations were considered eligible when they addressed biomaterials used for mastoid obliteration or closely related mastoid/temporal bone reconstruction models [14,15,16,17]. The review protocol was registered on the Open Science Framework (OSF) and is available at https://doi.org/10.17605/OSF.IO/752KG. 

The PRISMA 2020 checklist is provided in the Supplementary Materials.

The research question was: “What are the clinical outcomes, safety, and biological integration capability of biomaterials used for mastoid obliteration, and how do emerging synthetic materials, particularly bioactive glass, compare with conventional grafting techniques, based on both clinical and preclinical evidence?”

### 2.2. Inclusion Criteria and Eligibility

The study selection process was structured according to the PICOS framework (Population, Intervention, Comparator, Outcome, and Study design), in accordance with PRISMA 2020 recommendations [18,19]. Given the translational nature of the review question, eligibility criteria were defined separately for clinical and preclinical evidence while maintaining a common focus on biomaterials used for mastoid obliteration or mastoid/temporal bone reconstruction.

For the clinical synthesis, studies were considered eligible if they met the following criteria:

- Population: patients undergoing mastoid obliteration or mastoid reconstruction procedures in the context of otologic surgery, particularly following canal wall down mastoidectomy, canal wall up tympanomastoidectomy, revision mastoid surgery, or surgery for chronic otitis media and/or cholesteatoma.
- Intervention: use of biomaterials for mastoid cavity obliteration or reconstruction, including autologous materials (e.g., bone pâté, bone dust, cartilage, musculoperiosteal flaps), allogeneic grafts, and synthetic biomaterials such as hydroxyapatite, tricalcium phosphate, calcium phosphate-based materials, and bioactive glass. Emerging or biologically enhanced strategies, including composite scaffolds or growth factor-enhanced materials applied in human subjects, were also considered eligible [11,20].
- Comparator: comparison between different biomaterials, between obliterated and non-obliterated cavities, or between biomaterial-based reconstruction and conventional surgical approaches, when available.
- Outcome: reporting of at least one clinically relevant endpoint, including dry ear rate, infection control, epithelialization, cavity stability, cholesteatoma residual or recurrent disease, need for revision surgery, radiological findings, quality of life, hearing outcomes, or material-related outcomes such as resorption, degradation, exposure, extrusion, complications, and biological integration [10].
- Study design: randomized controlled trials, prospective or retrospective cohort studies, case–control studies, comparative clinical studies, and early-phase clinical or translational investigations evaluating innovative biomaterials in human subjects.

For the preclinical synthesis, experimental in vivo, in vitro, or combined in vivo/in vitro studies were considered eligible when they investigated biomaterials, scaffolds, bioactive molecules, growth factors, stem/stromal cells, or regenerative approaches in models relevant to mastoid obliteration, temporal bone reconstruction, bone regeneration, antibacterial activity, or biomaterial integration. These studies were not pooled with clinical studies but were analyzed separately to provide complementary mechanistic and translational insights into biomaterial performance [10,11].

Studies were excluded if they were unrelated to otologic surgery, did not involve mastoid obliteration or biomaterial-based cavity filling/reconstruction, or focused exclusively on non-otologic applications such as dental, maxillofacial, orthopedic, or implantology procedures without relevance to mastoid reconstruction. Additional exclusion criteria included case reports, conference abstracts, study protocols, systematic reviews and meta-analyses, letters, editorials, commentaries, book chapters, non-English publications, and studies without extractable clinical, experimental, or translational outcomes relevant to the objectives of this review.

This two-level eligibility strategy allowed for the integration of clinically relevant evidence on safety and surgical outcomes with preclinical evidence addressing biological mechanisms, scaffold behavior, osteoconduction, osteoinduction, antibacterial properties, and regenerative potential.

### 2.3. Search Strategy

A comprehensive literature search was performed across three electronic databases—PubMed, Scopus, and Web of Science—to identify studies investigating biomaterials used in mastoid obliteration. The search strategy combined controlled vocabulary terms and free-text keywords related to mastoid surgery (e.g., “mastoid obliteration”, “mastoid cavity”, “mastoidectomy cavity”, “mastoid reconstruction”) and biomaterials (e.g., “bone graft”, “biomaterials”, “bioactive glass”, “hydroxyapatite”, “tricalcium phosphate”), and was adapted to each database using the appropriate search fields (Title/Abstract for PubMed, TITLE-ABS-KEY for Scopus, and Topic Search (TS) for Web of Science) to ensure methodological consistency [18,21]. The database search was conducted on 16 May 2026. The search included studies published from January 1, 2010 to the date of the final search in order to capture evidence reflecting contemporary surgical techniques and recent developments in biomaterial science, particularly in the field of bioactive and multifunctional materials [5,11]. Only articles published in English were considered. No initial restrictions were applied with respect to study design in order to maximize the sensitivity of the search strategy [21]. The full search strings used for each database are reported in Table 1.

The search strategy was designed to ensure high sensitivity and to capture all relevant studies, including both clinical and preclinical investigations addressing biomaterial performance in mastoid obliteration [18,21]. Additionally, the reference lists of all included studies were manually screened to identify further relevant publications not retrieved during the database search, in accordance with established systematic review methodology [18].

### 2.4. Study Selection and Data Extraction

All records retrieved from the database searches were exported to reference management software, and duplicates were removed before screening. Study selection was performed in two sequential phases, consisting of title/abstract screening followed by full-text assessment, in accordance with PRISMA 2020 recommendations [18]. Two reviewers independently screened all records and assessed full texts according to the predefined eligibility criteria. Disagreements were resolved by discussion or, when necessary, by consultation with a third reviewer with expertise in biostatistics, to ensure consistency and minimize selection bias [21].

For each included study, data were extracted using a predefined framework including first author, year of publication, country, study design, sample size, population or experimental model, biomaterial type, surgical approach or experimental intervention, follow-up, and reported outcomes. Clinical endpoints included dry ear rate, infection control, epithelialization, cholesteatoma recurrence, revision surgery, hearing outcomes, quality of life, radiological findings, and material-related complications. Preclinical endpoints included biocompatibility, osteoconduction, osteoinduction, antibacterial activity, scaffold integration, bone regeneration, and histological or imaging-based outcomes [21].

To facilitate comparison across studies, the following operational definitions were adopted throughout this review whenever outcome definitions were not explicitly standardized by the original authors. A dry ear was defined as the absence of persistent otorrhea at follow-up. A stable cavity was defined as a well-epithelialized mastoid cavity without chronic inflammation, recurrent infection, or the need for frequent cleaning. Complete epithelialization referred to full epithelial coverage of the reconstructed cavity. Residual cholesteatoma indicated disease persisting after the index surgery, whereas recurrent cholesteatoma referred to new disease developing after apparently complete removal. Revision surgery was defined as any subsequent surgical procedure required because of recurrent disease, persistent cavity problems, or material-related complications.

### 2.5. Risk of Bias Assessment

Methodological quality and risk of bias were assessed using validated design-specific instruments according to study design.

Non-randomized clinical studies were evaluated using the Risk Of Bias In Non-randomized Studies of Interventions (ROBINS-I) tool [22]. ROBINS-I assesses bias across seven domains, including confounding, selection of participants, classification of interventions, deviations from intended interventions, missing data, measurement of outcomes, and selection of the reported results. Overall risk of bias was classified as low, moderate, serious, or critical according to ROBINS-I guidance.

Randomized controlled trials were assessed using the Cochrane Risk of Bias 2 (RoB 2) tool [23], which evaluates bias across five domains: the randomization process, deviations from intended interventions, missing outcome data, measurement of outcomes, and selection of the reported results. Overall judgments were categorized as low risk of bias, some concerns, or high risk of bias.

Preclinical studies were evaluated using SYRCLE’s risk-of-bias tool [24], which was specifically developed for animal studies and adapted from the Cochrane risk-of-bias framework. SYRCLE assesses multiple domains, including selection bias, performance bias, detection bias, attrition bias, and reporting bias.

All assessments were performed independently by two reviewers. Disagreements were resolved through discussion and, when necessary, consultation with a third reviewer. The results were interpreted qualitatively using a domain-based approach and were used to support the narrative synthesis of the evidence.

### 2.6. Methodological Quality Assessment

The methodological quality assessment was performed independently by two reviewers after completion of study selection. Any discrepancies were resolved through discussion or, when required, consultation with a third reviewer with expertise in biostatistics, in accordance with established methodological guidance for systematic reviews [21].

The results of the risk-of-bias assessments were used to support a qualitative, domain-based interpretation of methodological rigor across the included studies. Particular attention was given to sources of bias that could influence the validity and interpretability of the findings, including confounding, participant selection, outcome assessment, missing data, reporting transparency, randomization procedures, and blinding, when applicable.

Given the substantial methodological and clinical heterogeneity across the included studies, including differences in study design, patient populations, surgical approaches, biomaterial categories, outcome definitions, outcome assessment methods, and follow-up duration, a quantitative meta-analysis was not considered appropriate. In addition, several studies reported outcomes using non-comparable endpoints and did not provide sufficient statistical data to permit robust quantitative pooling. Therefore, findings were synthesized qualitatively, with clinical and preclinical evidence analyzed separately and subsequently integrated to provide a translational interpretation of biomaterial performance in mastoid obliteration.

To facilitate interpretation of the evidence and provide a concise overview of between-study variability, the principal sources of methodological and clinical heterogeneity identified among the included clinical studies are summarized in Table 2.

Methodological quality was not used as an exclusion criterion, but was considered during data interpretation and evidence synthesis in order to contextualize the strength and limitations of the available literature.

## 3. Results

The database search yielded a total of 405 records, including 104 from PubMed, 116 from Scopus, and 185 from Web of Science. In addition, four records were identified through manual screening of reference lists, resulting in a total of 409 records. After the removal of 199 duplicate records, 210 unique studies remained and were considered for screening.

All 210 records underwent title and abstract screening. At this stage, 23 articles were excluded based on publication type, including reviews, case reports, book chapters, and other non-original studies. A total of 187 records were therefore retained for further evaluation.

During the screening phase, 117 records were excluded as they were not relevant to mastoid obliteration or did not involve biomaterials for cavity filling or did not report outcomes of interest. Consequently, 70 studies were deemed potentially eligible and their full text was assessed.

**Figure 1:**
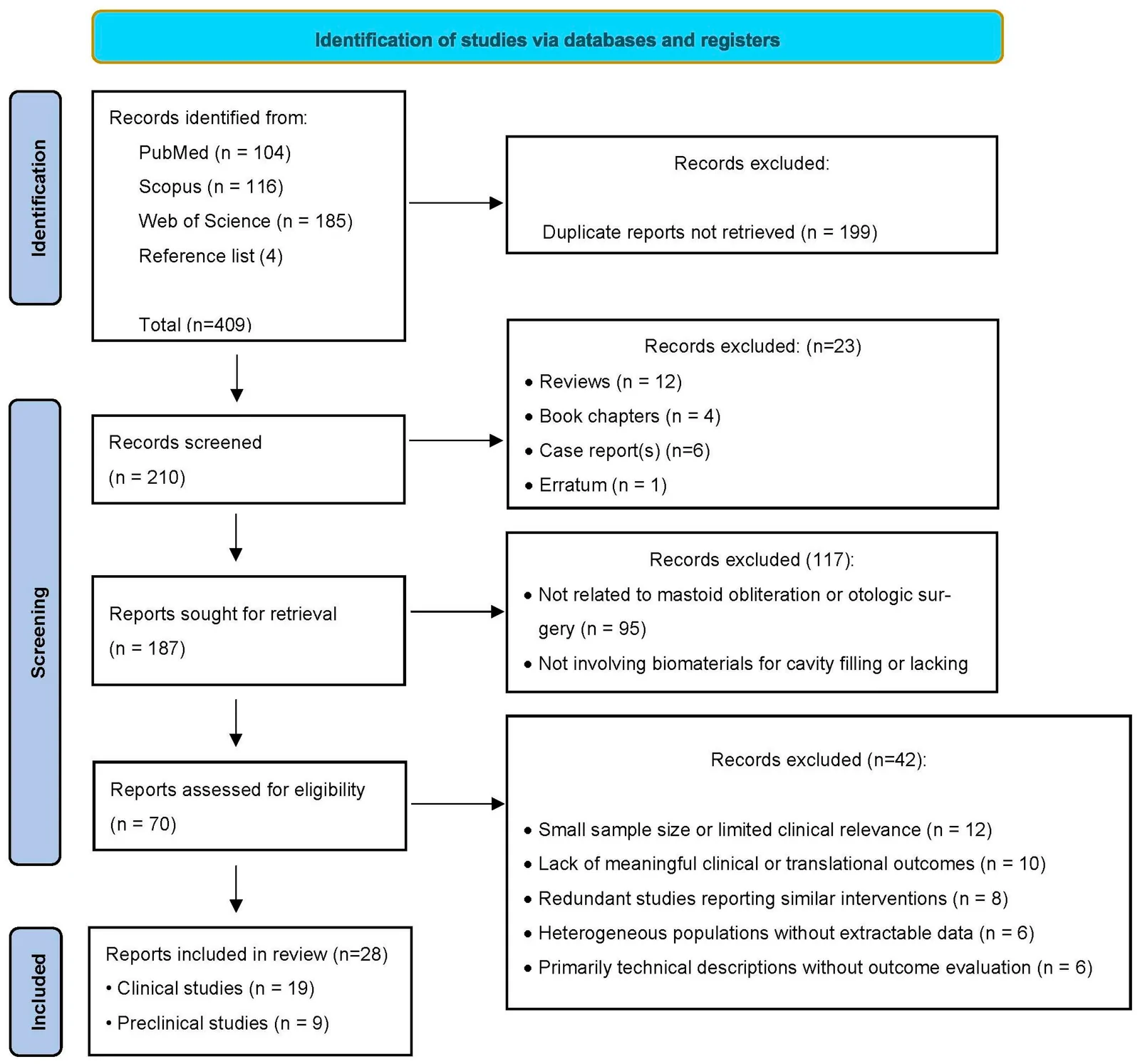
PRISMA flow diagram 2020. The flowchart displays the article search and selection process.

During full-text assessment, predefined eligibility criteria were applied to refine the pool of included studies and ensure the inclusion of clinically and methodologically relevant evidence. To ensure transparency and reproducibility of the selection process, explicit exclusion criteria were applied. Studies were excluded due to small sample size or limited clinical relevance (n = 12), lack of meaningful clinical or translational outcomes (n = 10), redundant studies reporting similar interventions (n = 8), heterogeneous populations without extractable data (n = 6), and primarily technical descriptions without outcome evaluation (n = 6). Detailed reasons for exclusion are summarized in Figure 1.

This additional screening step was implemented to enhance the overall quality and interpretability of the evidence, ensuring that only studies providing clinically or translationally relevant and non-redundant data were included in the final synthesis.

Ultimately, 28 studies met all eligibility criteria and were included in the qualitative synthesis, comprising 19 clinical studies and 9 preclinical studies. The study selection process is summarized in the PRISMA flow diagram in Figure 1.

To enhance the clarity and interpretability of the results, studies were further stratified into clinical and preclinical categories. When multiple studies reported similar interventions, those with the most robust methodology, largest sample size, and longest follow-up were prioritized to avoid redundancy. Only studies meeting the predefined eligibility criteria were included in the systematic synthesis, PRISMA counts, and risk-of-bias assessments. Additional references cited in the Discussion were used solely to provide contextual interpretation of specific clinical issues and were not considered part of the included study set.

The main characteristics of the included studies are summarized in Table 2 and Table 3. Clinical studies primarily focused on surgical outcomes and complication rates, whereas preclinical studies investigated the biological performance and regenerative potential of emerging biomaterials.

In Table 4, we reported the preclinical studies on emerging biomaterials for mastoid obliteration included in this review.

### Quality Assessment of Included Studies 

The methodological quality and risk-of-bias assessments are summarized in Table 4, Table 5 and Table 6. Overall, the included evidence demonstrated variable methodological rigor across both clinical and preclinical studies, reflecting substantial differences in study design, sample size, reporting quality, and methodological transparency. Among the clinical studies, the most frequent sources of bias identified through ROBINS-I were confounding and participant selection. These limitations were particularly evident in retrospective single-center studies and uncontrolled observational cohorts, where the absence of randomization and adjustment for potential confounders may have influenced treatment-effect estimates. Comparative cohort studies generally demonstrated lower overall risk of bias, although residual confounding remained a common methodological concern. Most studies showed low risk in the domains related to intervention classification, deviations from intended interventions, and selection of reported results. Only one study (Mishra et al. [36]) met the criteria for a randomized controlled trial and was therefore assessed using the Cochrane RoB 2 tool, whereas all remaining clinical studies were evaluated using ROBINS-I. The single randomized controlled trial assessed using the Cochrane Risk of Bias 2 tool demonstrated an acceptable methodological profile, with low risk of bias across most domains. Some concerns remained regarding the selection of reported results because no prospectively registered protocol or pre-specified statistical analysis plan was clearly available for verification.

Methodological quality among preclinical studies, assessed using SYRCLE’s risk-of-bias tool, was heterogeneous. Selection bias was generally low when random allocation procedures were reported; however, incomplete reporting of allocation concealment, housing procedures, and blinding of investigators or outcome assessors frequently resulted in unclear judgments across several domains. Detection bias and reporting bias were often difficult to assess because of insufficient methodological details, whereas attrition bias was generally low except in studies reporting animal losses or exclusions during follow-up. Overall, the methodological assessment supports cautious interpretation of the available evidence. While the included studies consistently reported favorable outcomes for mastoid obliteration across a wide range of biomaterials, the predominance of observational clinical studies and the limited methodological reporting in several experimental investigations reduce the overall certainty of the evidence. Consequently, further high-quality comparative studies, randomized trials, and rigorously designed translational investigations are needed to strengthen the evidence base in this field.

All 19 clinical studies were included in the risk of bias assessment. Overall, methodological quality showed variability across studies, with differences related to study design, reporting completeness, and control of confounding factors. Studies with larger sample sizes and comparative designs generally demonstrated higher methodological robustness. The most common limitations included lack of confounder identification and absence of adjustment strategies, particularly in smaller or retrospective studies.

In Table 7, we report the risk of bias assessment of the 9 preclinical studies selected.

The methodological quality of the preclinical studies showed considerable variability. Studies with clearly defined experimental designs and more complete reporting demonstrated greater methodological consistency, whereas limitations were primarily related to incomplete reporting of allocation procedures, lack of blinding, and variability in experimental design.

## 4. Discussion

### 4.1. Clinical Evidence

The clinical evidence included in this review supports mastoid obliteration as an effective surgical strategy for reducing the long-term morbidity associated with mastoidectomy, particularly after canal wall down procedures. Across the included studies, obliteration was consistently associated with improved cavity stability, reduced chronic discharge, better epithelialization, lower cavity-care burden, and, in selected cohorts, improved disease control. However, the overall interpretation of the clinical literature suggests that the major determinant of success may not necessarily be the intrinsic superiority of one material over another, but rather the effective elimination of the mastoid cavity and restoration of a more stable postoperative anatomy [25,26,27,28].

Autologous materials continue to represent one of the most established options for mastoid obliteration. Bone pâté, bone dust, cartilage, and musculoperiosteal flaps are biologically familiar to the host environment, inexpensive, readily available, and associated with favorable integration. Clinical studies using autologous materials have reported high rates of dry, stable, and epithelialized cavities, with low rates of persistent otorrhea and recurrent disease [33,34,37,38,39]. The reliability of these materials is likely related to their biocompatibility, limited foreign-body reaction, and capacity to support osteogenic and fibrous integration within the reconstructed mastoid space. Nevertheless, autologous grafts are not without limitations. Their performance may be influenced by the amount and quality of available tissue, surgical handling, resorption over time, and variability in the reconstruction technique. Moreover, autologous materials do not provide intrinsic antibacterial activity and may be less predictable in revision surgery or in chronically infected cavities.

Among synthetic materials, S53P4 bioactive glass has emerged as one of the most extensively investigated and clinically consistent alternatives. The studies included in this review suggest that S53P4 can provide durable cavity obliteration, favorable epithelialization, and good disease control in both canal wall down and selected canal wall up settings [25,26,27,40,51]. Its clinical appeal is based on the combination of structural support, osteoconductive behavior, and antibacterial properties, which may be particularly relevant in chronically infected or high-risk middle ear environments [16]. Long-term and comparative data indicate that S53P4 may achieve outcomes comparable to autologous materials, while offering a standardized off-the-shelf option and avoiding donor-site limitations [27,40,51]. However, available evidence does not conclusively demonstrate that bioactive glass is universally superior to autologous reconstruction. Rather, its main advantage appears to lie in its reproducibility, handling characteristics, volume stability, and biological activity in contaminated surgical fields.

Hydroxyapatite-based materials show more heterogeneous clinical results. Ready-to-use hydroxyapatite bone cement and selected hydroxyapatite formulations have been associated with satisfactory cavity reconstruction and anatomical stability [31,41]. Conversely, other studies have reported less predictable integration, higher revision rates, or complications related to material exposure, fragmentation, or incomplete biological incorporation, particularly with highly porous formulations [42]. These findings suggest that hydroxyapatite should not be considered a single uniform material category. Its clinical behavior is strongly influenced by formulation, porosity, resorption kinetics, mechanical properties, and the quality of soft-tissue coverage. Therefore, the variability observed across studies may reflect material-specific characteristics and surgical context rather than a general limitation of hydroxyapatite itself.

More recent clinical studies have explored biologically enhanced or composite approaches. Concentrated growth factors combined with hydroxyapatite have been associated with faster epithelialization and improved healing dynamics, supporting the concept that biological stimulation may enhance the performance of otherwise osteoconductive materials [30]. Similarly, sticky bone and other composite constructs represent attempts to combine structural stability with biological activity, although the available clinical evidence remains preliminary and based on limited sample sizes [29]. Allograft materials have also shown acceptable safety and clinical performance, but current evidence remains insufficient to establish their long-term reliability compared with autologous grafts or bioactive glass [32].

Importantly, comparative studies and reviews do not consistently demonstrate a clear superiority of a single biomaterial in terms of recurrence, dry ear rates, or overall complications [14,36,52,53]. This finding has important clinical implications. The success of mastoid obliteration appears to depend on a combination of factors, including complete disease removal, adequate reduction or elimination of the mastoid bowl, restoration of a stable epithelial surface, prevention of retraction-prone spaces, and careful long-term follow-up. In cholesteatoma surgery, the benefit of obliteration may therefore be mediated primarily by anatomical reconstruction and reduction of poorly ventilated recesses rather than by a material-specific effect alone [54]. From this perspective, the biomaterial should be selected according to surgical indication, cavity size, infection status, availability of autologous tissue, surgeon experience, need for radiological surveillance, and long-term reconstruction goals.

Concomitant reconstruction of the external auditory canal or posterior canal wall is an important, but inconsistently reported, component of mastoid obliteration. Among the included clinical studies, Luntz et al. reported canal wall down mastoidectomy with external auditory canal reconstruction in 127 ears [25], Hashmi et al. combined obliteration with posterior meatal wall reconstruction in 26 patients [31], and Fieux et al. reconstructed the posterior canal wall before obliteration in 32 patients [35]. These reconstructive procedures should be distinguished from enlargement procedures, such as meatoplasty or canaloplasty, which primarily facilitate inspection and cleaning of an open cavity. Because concomitant reconstruction or enlargement was not documented uniformly, and because the clinical cohorts included different canal wall down and canal wall up approaches, a pooled prevalence cannot be calculated reliably.

Hearing outcomes should likewise be interpreted separately from anatomical cavity control. In the randomized comparison by Mishra et al., the mean air–bone gap improved from 35.10 ± 5.21 dB preoperatively to 16.80 ± 4.23 dB at 12 months, without a significant difference between bone pâté and bioactive glass [36]. Meena et al. observed hearing improvement in all three obliteration groups after three months, although early differences were reported among muscle flap, bone dust, and hydroxyapatite [33]. In the study by Fieux et al., air–bone-gap closure was similar with allograft bone and 45S5 bioactive glass (12 and 11 dB, respectively) [35], whereas van Waegeningh et al. observed air-conduction improvement in 75% of ears undergoing ossicular reconstruction [38]. These results do not establish a consistent material-specific audiometric advantage because postoperative hearing also depends on ossicular reconstruction, middle-ear status, disease extent, and baseline hearing.

Postoperative hearing-aid rehabilitation represents an additional functional endpoint that is rarely reported by biomaterial-specific studies. Chronically discharging canal wall down cavities, a widened or irregular external auditory canal, altered canal resonance, inadequate earmold retention, acoustic feedback, and occlusion-related moisture may reduce tolerance of conventional hearing aids. Obliteration with reconstruction may improve prosthetic fitting predominantly by restoring a dry, stable, and more physiological canal rather than through a demonstrated acoustic effect of the filler itself. As contextual evidence outside the systematically included cohort, Geerse et al. reported that the ability to use a conventional hearing aid increased from 3% before surgery to 57% after mastoid obliteration and canal reconstruction in a cohort of 249 patients, while the proportion with a hearing-aid indication remained 67% [55]. No included study directly compared real-ear acoustics, earmold fitting, feedback, aided speech perception, or hearing-aid tolerance across obliteration materials; consequently, the influence of material stiffness or composition on postoperative sound-pressure transmission remains theoretical.

Postoperative follow-up burden was also reported heterogeneously and should be distinguished from the total duration of surveillance. Meena et al. scheduled assessments at one and three months [33], and Nikam et al. evaluated outcomes at six months [34], whereas Luntz et al. scheduled annual otoscopy and non-echo-planar diffusion-weighted magnetic resonance imaging for up to four years [25]. Follow-up attendance in the latter study declined from 100% at the first annual visit to 67.2% among patients eligible for the fourth-year visit [25]. Complementary contextual evidence comparing revision surgery with and without obliteration demonstrated that regular cavity cleaning remained necessary in 82.1% and 100% of patients, respectively, but the median interval between outpatient cleaning sessions increased from six to 12 months after obliteration and canal-wall reconstruction [56]. Because most included studies did not report the actual number of visits per patient or standardized cleaning schedules, a pooled postoperative visit count cannot be determined.

The need for reoperation deserves consideration beyond the limitations of available follow-up. Indications include residual or recurrent cholesteatoma, persistent otorrhea, graft resorption, material exposure or extrusion, failure of epithelial coverage, canal stenosis, and complications requiring removal of the obliteration material. Fieux et al. reported revision in 6/15 patients (40.0%) treated with allograft bone compared with 1/17 patients (5.9%) treated with 45S5 bioactive glass [35]. In the hydroxyapatite-cement cohort reported by Hashmi et al., one of 26 patients (3.8%) required complete material explantation [31]. External contextual comparative evidence found reoperation rates of 14.3% after obliteration with canal-wall reconstruction and 17.4% after non-obliteration, without a significant between-group difference [56]. Revision of an obliterated mastoid may require re-exposure or removal of implanted material and careful identification of altered anatomical landmarks; however, comparative estimates of material-specific revision difficulty are unavailable. Non-echo-planar diffusion-weighted imaging remains important when direct inspection of the obliterated space is limited [25,35].

The duration of postoperative surveillance varied markedly across the included clinical evidence. Reported examples ranged from three months in Meena et al. [33] and six months in Nikam et al. [34] to 12 months in Mishra et al. [36], a mean of approximately 29 months in Sioshansi et al. [37], mean postoperative follow-up of 33 and 44 months in the 45S5 and allograft groups, respectively, in Fieux et al. [35], and a mean of 45 months in van Waegeningh et al. [38]. Luntz et al. scheduled follow-up for up to four years [25], whereas Kim et al. reported a mean follow-up of 39.2 months, with a range of 12–80 months [32]. Delille et al. additionally reported five-year Kaplan–Meier estimates, which should not be confused with complete five-year observation of every participant [26]. A separate contextual series not included in the systematic synthesis described 173 adults treated with S53P4, had a mean follow-up of 53 months, and involved surveillance extending up to 10 years [40]. Therefore, although follow-up approaching 6.7 years was documented among the included cohorts, genuinely long-term evidence remains limited and protocol-dependent.

### 4.2. Preclinical Evidence and Translational Perspective

The preclinical evidence provides important mechanistic insights that help interpret the variability observed in clinical outcomes. The available preclinical evidence suggests that conventional biomaterials used for mastoid obliteration, including hydroxyapatite and calcium phosphate-based substitutes, predominantly act as osteoconductive scaffolds.

They can support bone ingrowth and provide a framework for tissue integration, but they generally lack strong intrinsic osteoinductive capacity unless combined with biological signals [48,49,57]. This distinction is critical, as successful cavity filling does not necessarily imply true bone regeneration. A material may provide stable volume replacement without inducing organized, vascularized, and functionally integrated bone formation.

Experimental studies have progressively shifted from simple cavity filling toward scaffold-based regenerative strategies. Three-dimensional composite scaffolds, including constructs based on PCL, tricalcium phosphate, collagen, gelatin, hydroxyapatite, and bioactive glass, have been designed to improve mechanical stability, porosity, cellular infiltration, vascular ingrowth, and spatial control of new bone formation [43,44,50]. These studies suggest that scaffold architecture is a key determinant of regenerative performance. Pore size, interconnectivity, degradation rate, surface chemistry, and mechanical compatibility with host bone may influence the balance between fibrous encapsulation, osteoconduction, and effective osteogenesis.

The incorporation of bioactive molecules represents a further step toward biologically active mastoid reconstruction. Bone morphogenetic protein-2 (BMP-2), either incorporated into calcium-based scaffolds or combined with composite biomaterials, has been shown to enhance osteogenesis and accelerate new bone formation in experimental models [43,46]. These findings support the hypothesis that osteoconductive scaffolds may benefit from osteoinductive stimulation to achieve more complete and predictable bone regeneration. However, the clinical translation of growth factor-based strategies remains limited by concerns regarding dosing, release kinetics, cost, safety, local inflammatory response, and regulatory constraints.

Stem cell-based approaches further expand the regenerative potential of mastoid obliteration. Experimental models using mesenchymal stem/stromal cells or the stromal vascular fraction combined with scaffolds and blood-derived products have demonstrated enhanced bone formation and improved tissue integration compared with scaffold-only approaches [17,45,47]. These findings suggest that cell-based strategies may contribute to transforming mastoid reconstruction from passive obliteration into active tissue regeneration. Nevertheless, these approaches remain at an early translational stage. Major unresolved challenges include cell source, processing time, reproducibility, intraoperative feasibility, long-term safety, and the extent to which potential regenerative benefits justify procedural complexity.

Preclinical data also help explain the favorable clinical performance of S53P4 bioactive glass. Beyond osteoconduction, S53P4 has demonstrated antibacterial and antibiofilm properties, which may be particularly relevant in chronically infected ears and revision surgery [58]. Imaging-based studies have also suggested active bone formation after implantation, supporting the concept that bioactive glass may participate in tissue remodeling rather than acting solely as an inert filler [59]. These biological properties provide a plausible mechanistic basis for its clinical use in high-risk environments. However, extrapolation from experimental and imaging data to clinical decision-making should be approached cautiously, as animal models and controlled laboratory conditions cannot fully reproduce the complexity of the human mastoid, including aeration patterns, microbiology, inflammatory status, and surgical variability.

Finally, experimental research on mastoid air cell regeneration introduces an even broader perspective. Rather than simply eliminating the mastoid cavity, future strategies may aim to restore a more physiological temporal bone structure, potentially combining reconstruction, aeration, mucosalization, and bone regeneration [60]. While this concept remains largely investigational, it highlights a potential evolution of the field from obliteration as a space-filling procedure toward reconstruction as a biologically guided restoration of form and function.

### 4.3. Future Research Priorities for Clinical Translation

Despite the promising results of emerging biomaterials and tissue-engineering approaches, several key unanswered questions must be addressed before these strategies can be routinely translated into clinical practice. Future research should address the following priorities: (1) which biomaterial characteristics, including porosity, degradation profile, mechanical properties, and scaffold architecture, are most strongly associated with long-term clinical success; (2) whether biologically enhanced constructs can provide clinically meaningful advantages over currently established materials such as autologous grafts and S53P4 bioactive glass; (3) how biological integration, vascularization, and bone regeneration can be objectively and reproducibly assessed in human studies; (4) what imaging and surveillance protocols are most appropriate for monitoring tissue integration while maintaining reliable detection of residual or recurrent cholesteatoma; (5) how regenerative constructs perform in the long term with respect to infection control, material stability, and revision surgery requirements; and (6) which standardized clinical, radiological, audiological, and patient-reported outcome measures should be adopted to improve comparability across future studies. Addressing these questions will be essential to bridge the gap between experimental innovation and routine clinical application.

### 4.4. Integrated Interpretation

Taken together, the clinical and preclinical evidence suggests a progressive evolution of mastoid obliteration through three conceptual phases. Unlike previous reviews that primarily focused on the clinical performance of individual obliteration materials [13,14,51,52,53], the present review integrates both clinical and preclinical evidence within a translational framework. This approach allows biomaterials to be interpreted not only according to their surgical outcomes, but also according to their biological activity and regenerative potential. The first phase is represented by traditional autologous obliteration, in which the primary goal is mechanical cavity reduction and stabilization. This approach appears to remain clinically effective and biologically reliable, particularly when adequate autologous tissue is available and disease clearance is complete [33,34,37,38,39]. The second phase is represented by bioactive synthetic materials, especially S53P4 bioactive glass, which introduce standardized availability, osteoconductive behavior, and antibacterial activity into the reconstructive strategy [25,26,27,40,51,58,59]. The third and most recent phase is represented by regenerative tissue engineering approaches, in which scaffolds are combined with growth factors, blood-derived products, or stem/stromal cells to promote active bone formation and tissue integration [17,43,44,45,46,47,48,49,50].

This integrated interpretation suggests that the key question is not simply which material fills the mastoid cavity most effectively, but which reconstructive strategy best matches the biological and clinical needs of each patient. In a dry, stable, primary surgical field, autologous materials may remain sufficient and highly reliable. In chronically infected or revision cases, bioactive glass may offer additional theoretical and practical advantages because of its antibacterial behavior and standardized handling. In the future, biologically enhanced scaffolds may become relevant when the goal extends beyond cavity obliteration toward true bone regeneration. However, the current clinical evidence does not yet support the routine use of advanced regenerative constructs outside selected or investigational contexts.

A further relevant point is the distinction between anatomical success and biological success. Anatomical success is achieved when the cavity becomes smaller, dry, stable, and self-cleaning. Biological success would imply predictable integration, vascularization, bone formation, resistance to infection, and long-term structural stability. Most clinical studies currently evaluate the former, whereas preclinical investigations predominantly address the latter. This mismatch may partly explain why clinical and experimental evidence are difficult to compare directly. Bridging this gap will require translational studies that combine clinically meaningful endpoints with objective measures of material integration, imaging-based assessment, and standardized long-term follow-up.

**Figure 2:**
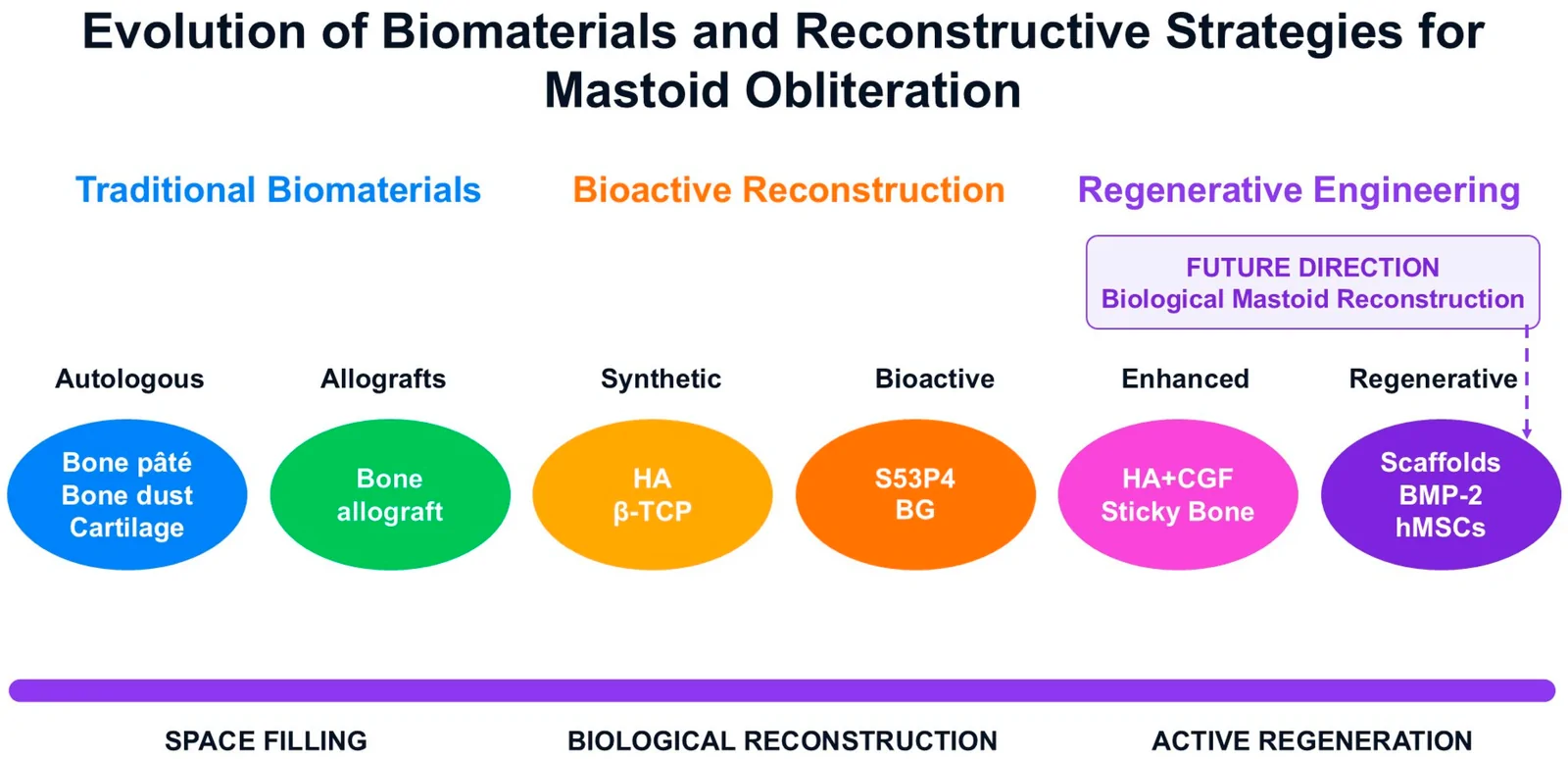
Evolution of biomaterials and reconstructive strategies for mastoid obliteration. The figure illustrates the transition from traditional cavity-filling materials to bioactive and regenerative approaches, highlighting the progression from space filling to biological reconstruction and active regeneration. Blue and green colors represent conventional biomaterials primarily used for structural cavity obliteration. Orange tones indicate bioactive materials designed to promote biological interaction and tissue integration. Purple tones represent advanced regenerative strategies aimed at stimulating tissue regeneration and biological mastoid reconstruction. The arrows illustrate the conceptual progression and future direction of the field toward increasingly regenerative approaches.

Overall, the findings of this review support mastoid obliteration as an effective surgical concept while also indicating that the choice of biomaterial should be interpreted within a broader reconstructive framework. Material selection should consider not only availability and handling, but also infection risk, expected integration, radiological follow-up, revision potential, and the biological goals of surgery. At present, autologous materials remain among the most established option, S53P4 bioactive glass represents one of the most consistent synthetic alternatives, and tissue engineering approaches define a promising but still insufficiently validated frontier. This conceptual framework extends beyond a simple comparison of individual biomaterials and provides a translational interpretation of the field, linking current clinical practice with emerging regenerative approaches. The progressive evolution from structural cavity filling to biological reconstruction and active regeneration is summarized in Figure 2.

Patient-reported outcomes further support the clinical relevance of mastoid obliteration beyond anatomical cavity control. Previous studies evaluating quality of life after cholesteatoma surgery have shown that canal wall down procedures combined with mastoid obliteration may achieve quality-of-life outcomes comparable to canal wall up techniques. In addition, revision canal wall down mastoidectomy with mastoid obliteration has been associated with favorable postoperative quality-of-life results, although residual symptoms related to hearing, balance, or cavity-related discomfort may persist in selected patients. These findings suggest that future studies should evaluate mastoid obliteration not only through otoscopic, radiological, and recurrence-related endpoints, but also through patient-centered outcomes such as quality of life, symptom burden, hearing-related complaints, water tolerance, and need for postoperative care [61,62,63].

The conceptual evolution of biomaterials and reconstructive strategies for mastoid obliteration is summarized in Figure 2.

### 4.5. Limitations

Several limitations should be acknowledged when interpreting the findings of this review. First, the clinical evidence remains heterogeneous in design and methodological quality. Furthermore, only one randomized controlled trial and 18 non-randomized clinical studies were identified, limiting the ability to draw strong comparative conclusions regarding the superiority of specific biomaterials.

Sample sizes, surgical indications, disease severity, revision status, follow-up duration, and reconstruction techniques vary substantially across studies. These factors limit the strength of direct comparisons and increase the likelihood that observed outcomes may be influenced by selection bias, surgeon expertise, or institutional protocols rather than by the biomaterial itself. In particular, several studies showed incomplete identification and control of confounding factors, as reflected in the domain-based methodological assessment.

Second, the included studies use heterogeneous definitions of success. Outcomes such as “dry ear,” “stable cavity,” “complete epithelialization,” “recurrence,” “residual disease,” and “revision surgery” are not always defined consistently. Follow-up duration is also variable, and this is particularly relevant in cholesteatoma surgery, where recidivism may occur years after the initial procedure. Short- and medium-term outcomes may therefore overestimate long-term effectiveness, especially in studies without systematic otoscopic and radiological surveillance. Inconsistent reporting of concomitant canal reconstruction or enlargement, postoperative visit frequency, hearing-aid use, material-specific acoustic measurements, and revision complexity also precluded pooled estimates for these outcomes.

Third, the biomaterials themselves are highly heterogeneous. Even within a single material category, such as hydroxyapatite or calcium phosphate, differences in formulation, porosity, granule size, cement properties, degradation rate, and combination with biological adjuncts may lead to substantially different clinical behavior. As a result, broad comparisons between “autologous,” “heterologous,” and “synthetic” materials may oversimplify material-specific performance. This limitation is particularly relevant when interpreting complications, integration, and long-term stability.

Fourth, the integration of clinical and preclinical evidence is inherently challenging. Preclinical studies provide valuable mechanistic data on osteogenesis, scaffold architecture, antibacterial activity, and tissue integration, but they often rely on small animal models that do not reproduce the anatomical complexity, aeration pathways, mucosal environment, chronic infection, and surgical variability of the human mastoid. In addition, many experimental studies show incomplete reporting of sample size, allocation procedures, blinding, or long-term outcomes, as reflected in the variability observed across the risk-of-bias domains. Therefore, while preclinical evidence is essential for understanding biological plausibility, it cannot be directly translated into clinical recommendations without further validation.

Finally, the qualitative nature of this synthesis limits the ability to quantify the relative effectiveness of individual materials. The heterogeneity of study designs, endpoints, and follow-up prevented robust pooled comparisons across all biomaterial classes. The restriction to English-language publications may also have introduced language-related publication bias. Future research should prioritize prospective multicenter studies, standardized outcome definitions, longer follow-up, systematic imaging protocols, and direct comparative designs. Translational studies should also aim to link biological endpoints, such as bone formation and material integration, with clinically meaningful outcomes, including cavity stability, recurrence, infection control, quality of life, and the need for revision surgery.

## 5. Conclusions

Mastoid obliteration appears to represent an effective and clinically relevant reconstructive strategy for improving outcomes after mastoidectomy, particularly by promoting cavity stabilization, reducing chronic otorrhea, facilitating epithelialization, and contributing to improved long-term control of cholesteatoma-related morbidity [33,34,37,39]. The evidence synthesized in this review suggests that the principal clinical benefit of obliteration is primarily related to anatomical reconstruction and elimination of the unstable mastoid cavity, whereas the selected biomaterial predominantly influences healing dynamics, biological integration, resistance to infection, and long-term structural behavior [14,53,54].

Autologous materials remain the most established and biologically reliable option because of their excellent biocompatibility, intrinsic osteogenic potential, availability, and reproducible clinical performance [33,34,37,39]. Among synthetic alternatives, S53P4 bioactive glass appears to be one of the most consistent biomaterials, combining osteoconductive properties with antibacterial and antibiofilm activity, which may be particularly advantageous in chronically infected, revision, or high-risk surgical fields [26,27,40,51,58,59]. Conversely, other materials, including hydroxyapatite-based formulations and allografts, show more variable outcomes, suggesting that their performance is highly dependent on material composition, porosity, resorption profile, handling characteristics, and the quality of surgical coverage and integration [31,32,41,42].

Preclinical evidence further supports a progressive shift from passive cavity filling toward biologically active reconstruction. Composite scaffolds, growth factor-enhanced constructs, blood-derived products, and stem cell-based strategies have demonstrated promising potential to enhance osteoconduction, osteoinduction, vascularization, and tissue integration within experimental mastoid models [17,30,43,45,46,47,50]. However, these approaches remain at an early translational stage, and their clinical superiority over established techniques has not yet been demonstrated.

Overall, mastoid obliteration should be interpreted not simply as a material-dependent procedure, but as a reconstructive strategy in which successful outcomes result from the integration of complete disease removal, stable anatomical cavity reduction, adequate epithelial coverage, infection control, and predictable biomaterial integration. At present, autologous materials and S53P4 bioactive glass represent the most clinically supported options, whereas advanced tissue engineering strategies define a promising but still insufficiently validated frontier.

A distinctive aspect of the present review is the integration of clinical and preclinical evidence into a unified translational framework, highlighting the evolution of mastoid obliteration from a volume-reduction procedure toward biologically active reconstruction and regenerative tissue-engineering strategies.

Future research should prioritize prospective comparative studies, standardized outcome definitions, longer clinical and radiological follow-up, and translational models capable of linking biological endpoints of regeneration with clinically meaningful outcomes, including dry ear rate, recurrence, infection control, quality of life, and the need for revision surgery.

## Figures and Tables

**Table 1:** Database, search strings and number of articles found.

Database	Search Strings	Number of Results
PubMed	((“mastoid obliteration” [Title/Abstract] OR “mastoid cavity”[Title/Abstract] OR “mastoidectomy cavity”[Title/Abstract] OR “mastoid reconstruction”[Title/Abstract]) AND (“bone graft”[Title/Abstract] OR biomaterial*[Title/Abstract] OR autologous[Title/Abstract] OR allogeneic[Title/Abstract] OR allogenic[Title/Abstract] OR synthetic[Title/Abstract] OR “bioactive glass”[Title/Abstract] OR hydroxyapatite[Title/Abstract] OR “tricalcium phosphate”[Title/Abstract] OR “calcium phosphate”[Title/Abstract] OR “bone substitute”[Title/Abstract] OR “bone pate”[Title/Abstract] OR “bone paste”[Title/Abstract])) NOT (dental[Title/Abstract] OR maxillofacial[Title/Abstract] OR implantology[Title/Abstract]) AND (“2010/01/01”[Date-Publication]: “3000”[Date-Publication]) AND (english[Language])	104
Scopus	TITLE-ABS-KEY (“mastoid obliteration” OR “mastoid cavity” OR “mastoidectomy cavity” OR “mastoid reconstruction”) AND TITLE-ABS-KEY (“bone graft” OR biomaterial* OR autologous OR allogeneic OR synthetic OR “bioactive glass” OR hydroxyapatite OR “tricalcium phosphate” OR “calcium phosphate” OR “bone substitute”) AND PUBYEAR > 2009 AND NOT TITLE-ABS-KEY (dental OR maxillofacial OR implant *)	116
Web of Science	(“mastoid obliteration” OR “mastoid cavity” OR “mastoidectomy cavity” OR “mastoid reconstruction”) AND (“bone graft” OR biomaterial* OR autologous OR allogeneic OR allogenic OR synthetic OR “bioactive glass” OR hydroxyapatite OR “tricalcium phosphate” OR “calcium phosphate” OR “bone substitute” OR “bone pate” OR “bone paste”) NOT (dental OR maxillofacial OR implant *)	185

**Table 2:** Main sources of heterogeneity across included clinical studies.

Domain	Heterogeneity Identified
Study design	Retrospective cohort studies, prospective studies, comparative studies, and one randomized controlled trial
Patient population	Primary cholesteatoma, recurrent or residual cholesteatoma, chronic suppurative otitis media, and revision mastoid surgery cases
Surgical approach	Canal wall down (CWD), canal wall up (CWU), and procedures combined with canal wall or external auditory canal reconstruction
Biomaterial category	Autologous grafts (bone pâté, bone dust, cartilage, musculoperiosteal flaps), allografts, hydroxyapatite-based materials, S53P4/45S5 bioactive glass, and biologically enhanced or composite constructs
Outcome assessment	Dry ear rate, cavity stability, epithelialization, cholesteatoma recurrence, hearing outcomes, quality of life, revision surgery, and radiological findings
Follow-up duration	Follow-up periods ranging from 3 to 88 months
Imaging surveillance	Variable use of otoscopic follow-up and radiological monitoring, including MRI surveillance in selected studies
Outcome definitions	Non-uniform definitions of dry ear, stable cavity, epithelialization, recurrence, and treatment success across studies

**Table 3:** Characteristics of the included clinical studies on biomaterials for mastoid obliteration.

First Author, Country, Journal, Year	Aim of the Study	(1) Study Design, (2) Sample Size, (3) Population	Conclusions
Luntz M. et al. [25], Israel, *Eur Arch ORL*, 2026	Assess long-term outcomes and recidivism after CWD mastoidectomy with EAC reconstruction, tympanoplasty, and S53P4 obliteration.	(1) Retrospective cohort study; (2) 127 ears; (3) Extensive cholesteatoma patients treated with CWD mastoidectomy, EAC reconstruction, tympanoplasty, and S53P4 obliteration.	High rate of dry, epithelialized ears (96.9%). Recidivism remained possible, supporting long-term clinical and MRI follow-up.
Delille et al. [26], France, *Otology & Neurotology*, 2026	Evaluate the effect of S53P4 bioactive glass obliteration on cholesteatoma recurrence after CWU surgery.	(1) Retrospective comparative cohort study; (2) 172 ears; (3) Patients undergoing CWU tympanomastoidectomy for cholesteatoma, with or without S53P4 obliteration.	S53P4 obliteration reduced cholesteatoma recurrence and improved disease-free survival, supporting its use as an adjunct to CWU surgery.
Kroon et al. [27], The Netherlands, *Otology & Neurotology*, 2025	Compare mastoid obliteration with S53P4 bioactive glass versus mastoidectomy alone in refractory CSOM	(1) Retrospective comparative cohort study; (2) 208 cases (124 obliteration vs 84 non-obliteration); (3) Refractory CSOM patients.	Higher dry-ear rate with obliteration (94% vs. 85%) and reduced need for revision surgery compared with mastoidectomy alone; supports S53P4 as an effective adjunct in refractory CSOM.
Chomarat et al. [28], France, *Eur Arch ORL*, 2025	To evaluate the effectiveness of bone-based mastoid obliteration in cholesteatoma surgery by comparing residual and recurrence rates across different surgical techniques and materials.	(1) Retrospective cohort study; (2) n = 236 surgeries; (3) Patients undergoing CWU, CWD, or CWD with reconstruction tympanoplasty for primary, recurrent, or residual cholesteatoma, with or without mastoid obliteration.	Bone pâté obliteration was associated with significantly lower residual and recurrence rates than non-obliterated cavities. No significant benefit was observed for G45S5 bioactive glass. Results support autologous bone obliteration in cholesteatoma surgery.
Zwierz et al. [29], Poland, *J Clin Med*, 2025	Compare sticky bone and S53P4 bioactive glass for mastoid obliteration after canal wall down surgery.	(1) Prospective preliminary study; (2) 28 patients (21 sticky bone; 7 S53P4); (3) Adults undergoing canal wall down mastoidectomy for chronic otitis media with cholesteatoma.	Both techniques showed comparable overall outcomes; however, the sticky bone approach demonstrated more favorable healing characteristics and fewer postoperative complications, suggesting it may represent a promising alternative to S53P4 bioactive glass for mastoid obliteration
Liu et al. [30], China, *Braz J Otorhinolaryngol*, 2025	Evaluate the effect of concentrated growth factors (CGF) combined with hydroxyapatite on epithelialization after CWD mastoidectomy.	(1) Retrospective cohort study; (2) 56 patients; (3) Patients undergoing CWD mastoidectomy for cholesteatoma treated with HA alone or HA + CGF.	CGF combined with hydroxyapatite accelerated epithelialization and improved healing compared with hydroxyapatite alone.
Hashmi et al. [31], UK, *J Laryngol Otol*, 2025	To evaluate the clinical outcomes of mastoid obliteration and posterior canal wall reconstruction using ready-to-use hydroxyapatite bone cement following canal wall down mastoidectomy.	(1) Retrospective cohort study; (2) 26 patients; (3) Patients undergoing CWD mastoidectomy with primary mastoid obliteration and posterior meatal wall reconstruction using hydroxyapatite bone cement.	Hydroxyapatite bone cement achieved excellent cavity stability and low complication rates, supporting its use for mastoid obliteration and canal wall reconstruction.
Kim et al. [32], Korea, *J Audiol Otol*, 2024	To evaluate the safety and effectiveness of bone allografts as a material for mastoid obliteration after mastoidectomy.	(1) Retrospective cohort study; (2) n = 76 ears (23 mastoid obliteration vs. 53 canal wall down without obliteration); (3) patients undergoing mastoidectomy for chronic otitis media with or without obliteration using bone allografts	Bone allografts showed favorable safety and clinical performance. No graft shrinkage or persistent otorrhea was reported in the obliteration group, and complication rates tended to be lower than in non-obliterated cavities. These findings support bone allografts as a potentially reliable option for mastoid obliteration.
Meena et al. [33], India, *Indian J Otolaryngol*, 2024	To compare the surgical outcomes and efficacy of different materials used for mastoid cavity obliteration following canal wall down mastoidectomy.	(1) Prospective comparative non-randomized study; (2) n = 60 patients; (3) individuals undergoing canal wall down mastoidectomy for cholesteatoma, divided into three groups according to obliteration material: muscle flap, bone dust, and hydroxyapatite.	Mastoid obliteration improved postoperative outcomes overall, with earlier and better cavity healing observed in the muscle flap and bone dust groups compared to hydroxyapatite. All techniques resulted in hearing improvement and reduced postoperative complications, but autologous materials demonstrated superior epithelialization and clinical outcomes.
Nikam et al. [34], India, *Indian J Otolaryngol*, 2023	To evaluate the effectiveness of different autologous materials used for mastoid obliteration in achieving a dry and epithelialized mastoid cavity after canal wall down mastoidectomy.	(1) Prospective non-randomized comparative study; (2) n = 48 patients; (3) individuals undergoing canal wall down mastoidectomy for cholesteatoma, divided into obliterated and non-obliterated groups; obliteration used autologous materials (bone dust, musculoperiosteal flap, or cartilage).	Mastoid obliteration using autologous materials is effective in achieving a dry, stable, and well-epithelialized cavity. Among the techniques, musculoperiosteal flap showed superior outcomes in terms of epithelialization and reduction in postoperative discharge compared to other materials.
Fieux et al. [35], France, *Sci Rep*, 2023	To compare clinical and anatomical outcomes of different obliteration materials, specifically allograft bone versus bioactive glass, in canal wall down mastoidectomy reconstruction.	(1) Retrospective comparative cohort study; (2) n = 32 patients: 15 treated with bone allograft and 17 with bioactive glass (45S5), (3) Patients undergoing canal wall down mastoidectomy with mastoid obliteration and reconstruction.	Bioactive glass (45S5) demonstrated superior clinical performance compared with allograft bone, with lower rates of postoperative otorrhea (5.9% vs. 53.3%) and revision surgery (5.9% vs. 40.0%). Audiological outcomes were similar, but bioactive glass showed advantages in maintaining cavity obliteration and reducing complications.
Weiss et al. [15], Germany, *Eur Arch ORL*, 2020	To evaluate the impact of mastoid cavity obliteration on hearing outcomes and health-related quality of life in patients with persistent mastoid cavities after canal wall down mastoidectomy.	(1) Prospective study; (2) n = 25 patients (25 ears); (3) patients undergoing revision canal wall down surgery with mastoid cavity obliteration using autologous materials; mean follow-up: 9.2 months.	Mastoid obliteration resulted in a significant improvement in health-related quality of life, with a marked reduction in symptom burden. A modest but statistically significant improvement in air conduction thresholds was observed, although quality of life improvements were not directly correlated with audiometric changes.
Mishra et al. [36], India, *J Laryngol Otol*, 2021	To compare the efficacy of bone pâté versus bioactive glass in mastoid obliteration following canal wall down mastoidectomy.	(1) Prospective randomized controlled trial; (2) n = 68 patients; (3) patients undergoing single-stage canal wall down mastoidectomy with mastoid obliteration: (a) Bone pâté group: n = 35 (b) Bioactive glass group: n = 33	Both materials demonstrated comparable effectiveness, with a high rate of dry, well-epithelialized cavities (≈95.6%) and significant hearing improvement. No differences were observed between groups in terms of healing, recurrence, or functional outcomes, although bioactive glass was associated with shorter operative time.
Sioshansi et al. [37], USA, *Otol Neurotol*, 2021	To evaluate the effectiveness and safety of mastoid obliteration using autologous bone dust following canal wall down mastoidectomy.	(1) Retrospective cohort study; (2) n = 43 ears; (3) patients undergoing canal wall down mastoidectomy with mastoid obliteration using autologous bone dust; mean follow-up about 29 months.	Mastoid obliteration with autologous bone dust achieved high rates of dry and stable cavities (95%), with low recurrence rates (5%). The technique was found to be safe, effective, and associated with reduced cavity-related morbidity and good long-term viability of the grafted material.
van Waegeningh et al. [38], The Netherlands, *Eur Arch ORL*, 2021	To evaluate safety, hygiene, and hearing outcomes of canal wall up surgery with bony mastoid obliteration (CWU-BOT) in patients with cholesteatoma, and to compare tympanic membrane reconstruction techniques.	(1) Retrospective cohort study; (2) n = 61 ears (44 primary cholesteatoma, 17 revision cases); (3) patients undergoing canal wall up tympanomastoidectomy with bony mastoid obliteration (bone chips and bone pâté); mean follow-up 45 months.	Bony mastoid obliteration in canal wall up surgery demonstrated a high level of safety and effectiveness, with a very low recurrence rate (3%) and no residual disease. Hearing improvement was observed in the majority of cases undergoing ossicular reconstruction (75%), supporting the reliability of this technique in achieving both durable disease control and favorable functional outcomes.
Kim BG et al. [39], South Korea, *Clinical and Experimental Otorhinolaryngology*, 2019	To evaluate clinical outcomes, hearing results, and postoperative complications of modified canal wall down mastoidectomy (mCWDM) with mastoid obliteration using autologous materials.	(1) Retrospective single-surgeon clinical study; (2) 76 patients; (3) patients with chronic otitis media (n = 22), cholesteatoma (n = 30), and adhesive otitis (n = 24) treated with mCWDM and mastoid obliteration using autologous crushed cartilage, bone pâté, and an inferior-based flap; mean follow-up: 64 months (20–89 months).	No recurrent/residual disease or cavity-related complications were observed. Hearing improved significantly, with only minor complications. mCWDM with autologous mastoid obliteration was considered safe and effective.
Kroon VJ et al. [40], The Netherlands, *Otology & Neurotology*, 2022	To evaluate the long-term safety and effectiveness of mastoid obliteration using S53P4 bioactive glass (BAG) in cholesteatoma surgery, focusing on recidivism, complications, hearing outcomes, and MRI-controlled follow-up.	(1) Retrospective cohort study; (2) 173 cases (171 adult patients); (3) Adult patients with cholesteatoma undergoing primary or revision CWU or CWD tympanomastoidectomy with mastoid obliteration using S53P4 bioactive glass between 2011 and 2021; mean postoperative follow-up: 53 months.	S53P4 bioactive glass proved to be a safe and effective material for mastoid obliteration, showing a low cholesteatoma recidivism rate (10%), excellent cavity outcomes (95% Merchant grade 0–1), absence of persistently wet ears, and preserved hearing without inner-ear toxicity.
Lindeboom JJ et al. [41], The Netherlands, *European Archives of Oto-Rhino-Laryngology*, 2023	To compare the efficacy and safety of hydroxyapatite versus bone pâté as mastoid obliteration materials in patients undergoing mastoidectomy for cholesteatoma or chronic suppurative otitis media.	(1) Retrospective multicenter cohort study; (2) 83 ears (45 hydroxyapatite, 38 bone pâté); (3) cholesteatoma (61 ears) or chronic suppurative otitis media (22 ears); primary/revision CWU/CWD mastoidectomy with obliteration; mean follow-up: 24-25 months; outcomes: complications, recidivism, infection control, and hearing.	Both hydroxyapatite and bone pâté provided safe and effective mastoid obliteration, with low recidivism, good infection control, and improved hearing. No major complications were observed; hydroxyapatite was associated with fewer postoperative wound infections.
Weiss NM et al. [42], Germany, *Otology & Neurotology*, 2020	To assess long-term clinical, radiological, audiological, and quality-of-life outcomes after open mastoid cavity obliteration using a highly porous hydroxyapatite matrix material (HMM).	(1) Cross-sectional cohort study with long-term follow-up; (2) 23 patients; (3) Cholesteatoma patients undergoing primary/revision tympanomastoid surgery with HMM mastoid obliteration; mean follow-up: 88.3 ± 21.4 months. Assessed outcomes: hearing, CT/MRI, recurrence, revision surgery, cavity status, and QoL (ZCMEI-21, GBI).	Air–bone gap improved significantly, but long-term outcomes were poor: revision surgery in 74% (17/23), recurrent cholesteatoma in four patients, and adequate cavity obliteration in only 39% of cases. Radiological surveillance was hindered by difficulty distinguishing recurrence from granulation/fibrous tissue. The authors do not recommend HMM for mastoid obliteration because of poor long-term obliteration rates, high revision rates, and surveillance limitations.

**Table 4:** Characteristics of the included preclinical studies on emerging biomaterials for mastoid obliteration.

First Author Country Journal, Year	Aim of the Study	(1) Study Design (2) Sample Size (3) Population	Conclusions
Park et al. [17], Korea, *Polymers*, 2022	To evaluate the potential of PCL-based scaffolds combined with stromal vascular fraction cells and autologous blood products to promote mastoid bone regeneration.	(1) Preclinical experimental study; (2) n = 20 rats divided into five groups; (3) rat mastoid bone defect model with implantation of PCL scaffolds with or without stromal vascular fraction cells and blood-derived growth factors; 7-month follow-up.	The combination of stromal vascular fraction cells with PCL scaffolds and blood-derived growth factors significantly enhanced new bone formation in mastoid defects, suggesting a promising strategy for one-step mastoid reconstruction based on tissue engineering approaches.
Yu et al. [43], China, *Regen Ther*, 2022	To develop and evaluate a 3D-printed bioactive scaffold combining S53P4 bioactive glass, polycaprolactone (PCL), and BMP-2 for mastoid obliteration and external auditory canal reconstruction.	(1) Preclinical experimental study; (2) n = 12 New Zealand rabbits simulating canal wall down mastoidectomy (4 groups, n = 3 per group); (3) rabbit canal wall down mastoidectomy model with implantation of 3D-printed S53P4/PCL scaffolds with or without BMP-2.	The 3D-printed S53P4/PCL scaffold demonstrated good biocompatibility, antibacterial activity, and significant osteogenic potential, enabling effective repair of mastoid defects and reconstruction of the external auditory canal in an animal model. The addition of BMP-2 further enhanced bone regeneration, suggesting potential for future clinical application.
Lee et al. [44], Korea, *Theranostics*, 2022	To develop and evaluate a highly elastic 3D-printed gelatin/hydroxyapatite scaffold loaded with human placental extract to enhance osteogenesis and bone regeneration in a mastoid obliteration model.	(1) Preclinical experimental in vivo and in vitro study; (2) n = 12 Sprague-Dawley rats (6 G/H and 6 G/H/hPE); (3) rat mastoid obliteration model with implantation of 3D-printed gelatin/hydroxyapatite scaffolds with or without human placental extract; evaluation by micro-CT, histology, immunohistochemistry, immunofluorescence and osteogenic analyses.	The G/H/hPE scaffold demonstrated excellent biocompatibility, enhanced cell proliferation, increased osteogenic differentiation and upregulation of osteogenic markers in vitro. In the rat mastoid obliteration model, hPE-loaded scaffolds promoted greater bone formation, angiogenesis, and osteogenic activity compared with gelatin/hydroxyapatite scaffolds alone, suggesting their potential for bone tissue engineering and mastoid reconstruction.
Choi et al. [45], Korea, *ACS Applied Bio Materials*, 2020	To investigate whether human tonsil-derived mesenchymal stem cells (hTMSCs) combined with a hydroxyapatite-chitosan patch enhance osteogenesis and mastoid obliteration in postoperative temporal bone defects.	(1) Preclinical experimental in vivo study; (2) n = 28 Sprague-Dawley rats divided into four groups (Sham, n = 7; hTMSCs, n = 7; HAp-chitosan patch, n = 7; hTMSCs + HAp-chitosan patch, n = 7); (3) rat tympanic bulla/temporal bone defect model evaluated by CT imaging, histology, immunohistochemistry, SEM and EDS analyses at 12 weeks.	HAp-chitosan patch implantation significantly improved mastoid obliteration and new bone formation compared with sham controls or hTMSC administration alone. The combination of hTMSCs with the HAp-chitosan patch further enhanced osteogenesis, promoted angiogenesis, increased bone density and bone formation in both peripheral and central regions of the tympanic bulla, supporting the potential of stem cell-loaded biomaterial scaffolds for temporal bone reconstruction.
Jang et al. [46], Korea, *In Vivo*, 2016	To evaluate whether the addition of bone morphogenetic protein-2 (BMP-2) and platelet-rich plasma (PRP) enhances osteogenesis of biphasic calcium phosphate (BCP) scaffolds in a mastoid obliteration model.	(1) Preclinical experimental study; (2) n = 21 rats allocated into three groups (7 per group); (3) rat mastoid obliteration model comparing BCP scaffolds alone, BMP-2-loaded BCP scaffolds, and BMP-2+PRP-loaded BCP scaffolds.	The addition of BMP-2 significantly enhanced osteogenesis compared to BCP alone, demonstrating increased bone formation and mineralization. However, the combined use of BMP-2 and PRP did not show a significant additional synergistic effect over BMP-2 alone. These findings suggest that BMP-2 is an effective osteoinductive adjunct in mastoid obliteration, whereas the contribution of PRP remains uncertain.
Skoloudik et al. [47], Czech Rep., *Cell Transplant*, 2016	To investigate whether implantation of human multipotent mesenchymal stromal cells (hMSCs) combined with hydroxyapatite enhances bone regeneration in postoperative temporal bone defects following mastoid obliteration.	(1) Preclinical experimental study; (2) n = 20 guinea pigs with temporal bone/mastoid defect (4 pilot + 8 experimental + 8 control); (3) guinea pig temporal bone/mastoid defect model comparing hydroxyapatite scaffold alone versus hydroxyapatite scaffold seeded with hMSCs.	The implantation of hMSCs combined with hydroxyapatite significantly increased new bone formation and the proportion of immature bone compared to the scaffold alone. The study demonstrated enhanced osteogenesis and suggested that MSC-based therapies may improve mastoid reconstruction outcomes by promoting active bone regeneration.
Jang et al. [48], Korea, *Acta Otolaryngol*, 2014	To compare the osteoconductivity of biologic hydroxyapatite (BHA) and artificial hydroxyapatite (AHA) in an experimental mastoid obliteration model.	(1) Preclinical experimental in vivo study; (2) n = 20 Sprague-Dawley rats divided into two groups: BHA group (n = 10) and AHA group (n = 10); (3) rat bulla/mastoid obliteration model assessed by micro-CT, confocal microscopy and histological analysis at 12 weeks.	Neither BHA nor AHA showed evidence of resorption during the 12-week follow-up. However, BHA demonstrated greater osteoconductivity, enhanced new bone formation, and more extensive mineralization than AHA according to micro-CT, confocal microscopy and histological findings, supporting its potential use as a superior material for mastoid obliteration.
Park et al. [49], Korea, *Int J Pediatr Otorhinolaryngol*, 2011	To evaluate the applicability and effectiveness of different calcium phosphate formulations for temporal dorsal bulla (mastoid) obliteration.	(1) Preclinical experimental in vivo study; (2) n = 26 guinea pigs (4 normal controls, 6 non-obliterated controls, 8 Polybone-G, and 8 Polybone-P); (3) temporal dorsal bulla obliteration model comparing granular β-tricalcium phosphate/polyphosphate (Polybone-G) and powder-type β-tricalcium phosphate/polyphosphate (Polybone-P); radiological (micro-CT) and histopathological evaluation performed at 8 and 20 weeks postoperatively.	Granular calcium phosphate (Polybone-G) induced greater new bone formation, lower inflammatory response, and better integration than powder-type calcium phosphate (Polybone-P). The granular material was almost completely resorbed by 20 weeks, whereas powder-type material persisted and was associated with ongoing inflammation, suggesting that granular calcium phosphate is a more suitable material for mastoid obliteration.
Jang et al. [50], Korea, *Macromol Biosci*, 2013	To evaluate whether a three-dimensional composite scaffold consisting of polycaprolactone (PCL), β-tricalcium phosphate (β-TCP), and collagen nanofibers enhances osteogenesis in a mastoid obliteration model.	(1) Preclinical experimental in vivo and in vitro study; (2) n = 20 male guinea pigs randomly divided into two groups (Group A: PCL/β-TCP/collagen nanofiber scaffold, n = 10; Group B: PCL/β-TCP scaffold, n = 10); (3) temporal bulla (mastoid obliteration) model evaluated by micro-CT, stereomicroscopy, fluorescent microscopy, and histological analyses at 4 and 12 weeks postoperatively, together with MG63 osteoblast-like cell culture studies.	The PCL/β-TCP/collagen nanofiber scaffold significantly improved cell attachment and proliferation, accelerated early bone formation, and promoted more extensive osteogenesis than the PCL/β-TCP scaffold alone. Micro-CT, fluorescent microscopy, and histological analyses demonstrated superior new bone formation and scaffold integration in the collagen-containing construct, supporting its potential use for mastoid obliteration and bone tissue engineering.

**Table 5:** Methodological quality and risk of bias of included studies (ROBINS-I).

First Author (Year)	Confounding	Selection of Participants	Classification of Interventions	Deviations from Intended Interventions	Missing Data	Measurement of Outcomes	Selection of Reported Results	Overall ROBINS-I
Luntz et al. [25] (2026)	Serious	Moderate	Low	Low	Moderate	Low	Low	Serious
Delille et al. [26] (2026)	Moderate	Moderate	Low	Low	Moderate	Moderate	Low	Moderate
Kroon et al. [27] (2025)	Moderate	Moderate	Low	Low	Low	Low	Low	Moderate
Chomarat et al. [28] (2025)	Moderate	Moderate	Low	Low	Low	Low	Low	Moderate
Zwierz et al. [29] (2025)	Serious	Moderate	Low	Low	Low	Moderate	Low	Serious
Liu et al. [30] (2025)	Serious	Moderate	Low	Low	Low	Moderate	Low	Serious
Hashmi et al. [31] (2025)	Serious	Moderate	Low	Low	Low	Moderate	Low	Serious
Kim et al. [32] (2024)	Serious	Moderate	Low	Low	Low	Moderate	Low	Serious
Meena et al. [33] (2024)	Serious	Moderate	Low	Low	Low	Moderate	Low	Serious
Nikam et al. [34] (2023)	Serious	Moderate	Low	Low	Low	Moderate	Low	Serious
Fieux et al. [35] (2023)	Moderate	Moderate	Low	Low	Low	Low	Low	Moderate
Weiss et al. [15] (2020)	Serious	Moderate	Low	Low	Moderate	Low	Low	Serious
Sioshansi et al. [37] (2021)	Serious	Moderate	Low	Low	Low	Moderate	Low	Serious
van Waegeningh et al. [38] (2021)	Moderate	Low	Low	Low	Low	Low	Low	Moderate
Kim et al. [39] (2019).	Moderate	Low	Low	Low	Low	Moderate	Moderate	Moderate
Kroon et al. [40] (2022)	Moderate	Moderate	Low	Low	Moderate	Low	Moderate	Moderate
Lindeboom et al. [41] (2023)	Moderate	Moderate	Low	Low	Low	Low	Moderate	Moderate
Weiss et al. [42] (2020)	Moderate	Moderate	Low	Low	Low	Moderate	Moderate	Moderate

**Table 6:** Methodological quality and risk of bias of the included randomized study (RoB 2).

First Author (Year)	Randomization Process	Deviations from Intended Interventions	Missing Outcome Data	Measurement of Outcomes	Selection of Reported Results	Overall RoB 2
Mishra et al. [36] (2021)	Low	Low	Low	Low	Some concerns	Some concerns

**Table 7:** Methodological quality and risk-of-bias domains of included preclinical studies (SYRCLE).

First Author (Year)	Study Design (Sample Size)	Selection Bias	Performance Bias	Detection Bias	Attrition Bias	Reporting Bias	Key Methodological Considerations
Park et al. [17] (2022)	Animal in vivo study (n = 20 rats, 5 groups)	Low (randomization reported)	Unclear	High (no blinding reported)	High (animal loss reported)	Unclear	Attrition bias due to deaths; lack of blinding; possible impact on outcome validity
Yu et al. [43] (2022)	Animal in vivo (n = 12 rabbits, 4 groups)	Low	Unclear	Unclear	Low	Unclear	Randomization reported; no blinding
Lee et al. [44] (2022)	Animal in vivo study, n = 12 rats, 2 groups (control: n = 6; experimental: n = 6)	Unclear	Unclear	Unclear	Low (no losses reported)	Unclear	Poor methodological reporting; randomization and blinding not described
Choi et al. [45] (2020)	Animal in vivo study (n = 28 Sprague-Dawley rats; 4 groups: Sham, n = 7; hTMSCs, n = 7; HAp-chitosan patch, n = 7; hTMSCs + HAp-chitosan patch, n = 7)	Unclear	Unclear	Low	Low	Unclear	Blinded histopathological assessment; no reported animal losses; randomization procedures and allocation concealment not described.
Jang et al. [46] (2016)	Animal in vivo (n = 21 rats, 3 groups)	Low	Unclear	Unclear	Low	Unclear	Randomization reported; no blinding reported
Skoloudik et al. [47] (2016)	Animal in vivo study (n = 20 guinea pigs; analyzed n = 15)	Low	Unclear	Low	High	Unclear	Randomization reported; blinded pathologist; attrition (animal death + exclusions)
Jang et al. [48] (2014)	Animal in vivo study (n = 20 Sprague-Dawley rats; BHA group, n = 10; AHA group, n = 10)	Low	Unclear	Unclear	Low	Unclear	Random allocation reported; no blinding reported.
Park et al. [49] (2011)	Animal in vivo study (n = 26 guinea pigs; 4 normal controls, 6 non-obliterated controls, 8 Polybone-G, 8 Polybone-P)	Low	Unclear	Low	Low	Unclear	Animals were randomly allocated to experimental groups and histopathological specimens were evaluated by a blinded pathologist. No animal losses were reported. Allocation concealment, housing randomization, and blinding of investigators during treatment administration were not described.
Jang et al. [50] (2013)	Animal in vivo and in vitro study (n = 20 guinea pigs; Group A: PCL/β-TCP/collagen nanofiber scaffold, n = 10; Group B: PCL/β-TCP scaffold, n = 10)	Low (random division reported)	Unclear	High (no blinding reported)	Low (all animals accounted for; no losses reported)	Unclear	Random division into groups was described; however, allocation concealment, investigator blinding, and blinded outcome assessment were not reported. No attrition was documented.

## Data Availability

No new data were generated for this study. All analyzed data are included in the article.

## Supplementary Materials

The following supporting information can be downloaded at: https://www.mdpi.com/article/10.3390/jcm15186952/s1. PRISMA 2020 Checklist [18].

## Abbreviations

The following abbreviations are used in this manuscript:

BCP	Biphasic calcium phosphate
BMP-2	Bone morphogenetic protein-2
BG	Bioactive Glass
CGFs	Concentrated growth factors
CSOM	Chronic suppurative otitis media
CWD	Canal wall down
CWU	Canal wall up
HA	Hydroxyapatite
hMSCs	Human multipotent mesenchymal stromal cells
PCL	Polycaprolactone
PRISMA	Preferred Reporting Items for Systematic Reviews and Meta-Analyses
PRP	Platelet-rich plasma
QoL	Quality of life
WoS	Web of Science
β-TCP	Beta-tricalcium phosphate

## References

[1] Kurien G., Greeff K., Gomaa N., Ho A. (2013). Mastoidectomy and mastoid obliteration with autologous bone graft: A quality of life study. J. Otolaryngol. Head Neck Surg..

[2] Yung M., Tassone P., Moumoulidis I., Vivekanandan S. (2011). Surgical management of troublesome mastoid cavities. J. Laryngol. Otol..

[3] Moffat D.A., Gray R.F., Irving R.M. (1994). Mastoid obliteration using bone pâté. Clin. Otolaryngol. Allied Sci..

[4] Mahendran S., Yung M.W. (2004). Mastoid obliteration with hydroxyapatite cement: The Ipswich experience. Otol. Neurotol..

[5] O’Brien F.J. (2011). Biomaterials & scaffolds for tissue engineering. Mater. Today.

[6] Vacanti J.P., Langer R. (1999). Tissue engineering: The design and fabrication of living replacement devices for surgical reconstruction and transplantation. Lancet.

[7] Hench L.L. (2006). The story of Bioglass. J. Mater. Sci. Mater. Med..

[8] Bohner M. (2000). Calcium orthophosphates in medicine: From ceramics to calcium phosphate cements. Injury.

[9] Pittenger M.F., Mackay A.M., Beck S.C., Jaiswal R.K., Douglas R., Mosca J.D., Moorman M.A., Simonetti D.W., Craig S., Marshak D.R. (1999). Multilineage potential of adult human mesenchymal stem cells. Science.

[10] Taguchi T., Lopez M.J. (2021). An overview of de novo bone generation in animal models. J. Orthop. Res..

[11] Sun K.H., Choi C.H., Jang C.H. (2026). Mastoid obliteration using bioceramic scaffold after canal wall down mastoidectomy: A systematic review. Ceramics.

[12] Moher D., Liberati A., Tetzlaff J., Altman D.G., PRISMA Group (2009). Preferred reporting items for systematic reviews and meta-analyses: The PRISMA statement. BMJ.

[13] Choong K.W.K., Kwok M.M.K., Shen Y., Gerard J.M., Teh B.M. (2022). Materials used for mastoid obliteration and its complications: A systematic review. ANZ J. Surg..

[14] Skoulakis C., Koltsidopoulos P., Iyer A., Kontorinis G. (2019). Mastoid obliteration with synthetic materials: A review of the literature. J. Int. Adv. Otol..

[15] Weiss N.M., Bächinger D., Botzen J., Großmann W., Mlynski R. (2020). Mastoid cavity obliteration leads to a clinically significant improvement in health-related quality of life. Eur. Arch. Otorhinolaryngol..

[16] van Gestel N.A., Geurts J., Hulsen D.J., van Rietbergen B., Hofmann S., Arts J.J. (2015). Clinical applications of S53P4 bioactive glass in bone healing and osteomyelitic treatment: A literature review. Biomed. Res. Int..

[17] Park S.H., Kim H., Lee Y.Y., Kim Y.J., Jang J.H., Choo O.-S., Choung Y.-H. (2022). Development of intracorporeal differentiation of stem cells to induce one-step mastoid bone reconstruction during otitis media surgeries. Polymers.

[18] Page M.J., McKenzie J.E., Bossuyt P.M., Boutron I., Hoffmann T.C., Mulrow C.D., Shamseer L., Tetzlaff J.M., Akl E.A., Brennan S.E. (2021). The PRISMA 2020 statement: An updated guideline for reporting systematic reviews. BMJ.

[19] Richardson W.S., Wilson M.C., Nishikawa J., Hayward R.S. (1995). The well-built clinical question: A key to evidence-based decisions. ACP J. Club.

[20] Chan C.Y., Chan Y.M. (2012). Mastoid obliteration and reconstruction: A review of techniques and results. Proc. Singap. Healthc..

[21] Higgins J.P.T., Thomas J., Chandler J., Cumpston M., Li T., Page M.J., Welch V. (2019). Cochrane Handbook for Systematic Reviews of Interventions.

[22] Sterne J.A.C., Hernán M.A., Reeves B.C., Savović J., Berkman N.D., Viswanathan M., Henry D., Altman D.G., Ansari M.T., Boutron I. (2016). ROBINS-I: A tool for assessing risk of bias in non-randomised studies of interventions. BMJ.

[23] Sterne J.A.C., Savović J., Page M.J., Elbers R.G., Blencowe N.S., Boutron I., Cates C.J., Cheng H.Y., Corbett M.S., Eldridge S.M. (2019). RoB 2: A revised tool for assessing risk of bias in randomised trials. BMJ.

[24] Hooijmans C.R., Rovers M.M., de Vries R.B.M., Leenaars M., Ritskes-Hoitinga M., Langendam M.W. (2014). SYRCLE’s risk of bias tool for animal studies. BMC Med. Res. Methodol..

[25] Luntz M., Hod K., Barzilai R. (2026). Medical outcomes after canal wall-down mastoidectomy, external ear canal reconstruction, tympanoplasty, and mastoid obliteration for extensive cholesteatoma. Eur. Arch. Otorhinolaryngol..

[26] Delille H., Bernardeschi D., Di Bari M., Sterkers O., Tankéré F., Lahlou G., Alciato L. (2026). Does mastoid and epitympanic obliteration with S53P4 bioactive glass reduce cholesteatoma recidivism in canal-wall-up surgeries? A retrospective clinical study. Otol. Neurotol..

[27] Kroon V.J., Colnot D.R., Mes S.W., Borggreven P.A., van de Langenberg R., Quak J.J. (2025). Mastoid obliteration using S53P4 bioactive glass versus mastoidectomy alone for refractory chronic suppurative otitis media. Otol. Neurotol..

[28] Chomarat J., Fabre C., Schmerber S., Quatre R. (2025). Evaluation of the effectiveness of bone obliteration in cholesteatoma surgery. Eur. Arch. Otorhinolaryngol..

[29] Zwierz A., Staszak M., Scheich M., Domagalski K., Hackenberg S., Burduk P. (2025). A comparison of the sticky bone obliteration technique and obliteration using S53P4 bioactive glass after canal wall down ear surgery: A preliminary study. J. Clin. Med..

[30] Liu M., Zhang L., Zhang Q., Zeng N., Li S., Guo S., Zhao Y., Tang M., Yang Q. (2025). Concentrated growth factors promote epithelization in the mastoid obliteration after canal wall down mastoidectomy. Braz. J. Otorhinolaryngol..

[31] Hashmi S., Hussain S.Z.M., Matto O., Dewhurst S., Qayyum A. (2025). To evaluate the results of mastoid obliteration and reconstruction of posterior meatal wall after canal wall down mastoidectomy using ready-to-use, self-setting hydroxyapatite bone cement. J. Laryngol. Otol..

[32] Kim W.J., Park C., Sim S., Hong T.U., Park S.Y., Heo K.W. (2024). Safety and effectiveness of bone allografts for mastoid obliteration after mastoidectomy: A pilot study. J. Audiol. Otol..

[33] Meena S., Kumar R., Meena R.K. (2024). Comparative analysis of various materials used for mastoid cavity obliteration in canal wall down mastoid surgery. Indian J. Otolaryngol. Head Neck Surg..

[34] Nikam S., Vedi J., Chandankhede V., Ekhar V., Shelkar R. (2023). Comparative study of various techniques of mastoid obliteration following canal wall down mastoidectomy. Indian J. Otolaryngol. Head Neck Surg..

[35] Fieux M., Tournegros R., Hermann R., Tringali S. (2023). Allograft bone vs. bioactive glass in rehabilitation of canal wall-down surgery. Sci. Rep..

[36] Mishra A.K., Mallick A., Galagali J.R., Gupta A., Sethi A., Ghotra A. (2021). Mastoid cavity obliteration using bone pâté versus bioactive glass granules in the management of chronic otitis media (squamous disease): A prospective comparative study. J. Laryngol. Otol..

[37] Sioshansi P.C., Alyono J.C., Blevins N.H. (2021). Mastoid obliteration using autologous bone dust following canal wall down mastoidectomy. Otol. Neurotol..

[38] van Waegeningh H.F., van Dinther J.J.S., Vanspauwen R., Zarowski A., Offeciers E. (2021). The bony obliteration tympanoplasty in cholesteatoma: Safety, hygiene and hearing outcome: Allograft versus autograft tympanic membrane reconstruction. Eur. Arch. Otorhinolaryngol..

[39] Kim B.G., Kim H.J., Lee S.J., Lee E., Lee S.A., Lee J.D. (2019). Outcomes of modified canal wall down mastoidectomy and mastoid obliteration using autologous materials. Clin. Exp. Otorhinolaryngol..

[40] Kroon V.J., Mes S.W., Borggreven P.A., van de Langenberg R., Colnot D.R., Quak J.J. (2022). Mastoid obliteration using S53P4 bioactive glass in cholesteatoma surgery: A 10-year single-center experience in 173 adult patients with long-term magnetic resonance imaging controlled follow-up. Otol. Neurotol..

[41] Lindeboom J.J., van Kempen P.M.W., Buwalda J., Westerlaken B.O., van Zuijlen D.A., Bom S.J.H., van der Beek F.B. (2023). Mastoid obliteration with hydroxyapatite vs. bone pâté in mastoidectomy surgery performed on patients with cholesteatoma and chronic suppurative otitis media: A retrospective analysis. Eur. Arch. Otorhinolaryngol..

[42] Weiss N.M., Stecher S., Bächinger D., Schuldt T., Langner S., Zonnur S., Mlynski R., Schraven S.P. (2020). Open mastoid cavity obliteration with a high-porosity hydroxyapatite ceramic leads to high rate of revision surgery and insufficient cavity obliteration. Otol. Neurotol..

[43] Yu F., Fan X., Wu H., Ou Y., Zhao X., Chen T., Qian Y., Kang H. (2022). Mastoid obliteration and external auditory canal reconstruction using 3D printed bioactive glass S53P4/polycaprolactone scaffold loaded with bone morphogenetic protein-2: A simulation clinical study in rabbits. Regen. Ther..

[44] Lee J., Kim D., Jang C.H., Kim G.H. (2022). Highly elastic 3D-printed gelatin/HA/placental-extract scaffolds for bone tissue engineering. Theranostics.

[45] Choi S.W., Kang J., Wang C., Lee H.M., Oh S.-J., Park K., Shin N., Lee I.-W., Lee J., Kong S.-K. (2020). Human tonsil-derived mesenchymal stem cells-loaded hydroxyapatite-chitosan patch for mastoid obliteration. ACS Appl. Bio Mater..

[46] Jang C.H., Choi C.H., Cho Y.B. (2016). Effect of BMP2-platelet-rich plasma-biphasic calcium phosphate scaffold on accelerated osteogenesis in mastoid obliteration. In Vivo.

[47] Skoloudik L., Chrobok V., Kalfert D., Koci Z., Sykova E., Chumak T., Popelar J., Syka J., Laco J., Dedková J. (2016). Human multipotent mesenchymal stromal cells in the treatment of postoperative temporal bone defect: An animal model. Cell Transplant..

[48] Jang C.H., Cho Y.B., Choi C.H., Jang Y.S., Jung W.K., Lee J.K. (2014). Comparison of osteoconductivity of biologic and artificial synthetic hydroxyapatite in experimental mastoid obliteration. Acta Otolaryngol..

[49] Park Y.H., Kim S.G., Lee J.W., Yoon Y.H. (2011). Obliteration of temporal dorsal bulla in guinea pigs using different types of calcium phosphate. Int. J. Pediatr. Otorhinolaryngol..

[50] Jang C.H., Cho Y.B., Yeo M.G., Kim G.H. (2013). Mastoid obliteration using three-dimensional composite scaffolds consisting of polycaprolactone/β-tricalcium phosphate/collagen nanofibers: An in vitro and in vivo study. Macromol. Biosci..

[51] Canali L., Di Bari M., Sheppard S.C., Alciato L., Sterkers O., Delille H., Bernardeschi D. (2026). Mastoid obliteration using granules of S53P4 bioactive glass: Systematic review and meta-analysis. Otolaryngol. Head Neck Surg..

[52] Viberti F., Monciatti G., Donniacuo A., Ferretti F., Salerni L., De Vito A., Bernardeschi D., Mandalà M. (2024). Heterologous materials are really better than autologous in tympanoplasty mastoid obliteration? A systematic review with meta-analysis. J. Int. Adv. Otol..

[53] Mendlovic M.L., Monroy Llaguno D.A., Schobert Capetillo I.H., Cisneros Lesser J.C. (2021). Mastoid obliteration and reconstruction techniques: A review of the literature. J. Otol..

[54] Bovi C., Luchena A., Bivona R., Borsetto D., Creber N., Danesi G. (2023). Recurrence in cholesteatoma surgery: What have we learnt and where are we going? A narrative review. Acta Otorhinolaryngol. Ital..

[55] Geerse S., Bost T.J.M., Allagul S., de Wolf M.J.F., Ebbens F.A., van Spronsen E. (2020). Hearing and hearing rehabilitation after obliteration of troublesome mastoid cavities. Eur. Arch. Otorhinolaryngol..

[56] van der Toom H.F.E., van der Schroeff M.P., Molenaar T.L., Metselaar M., van Linge A., Vroegop J.L., Pauw R.J. (2022). Revision surgery for chronically discharging mastoid cavities: Mastoid obliteration with canal wall reconstruction versus non-obliteration surgery. Eur. Arch. Otorhinolaryngol..

[57] Lee H.B., Lim H.J., Cho M., Yang S.-M., Park K., Park H.Y., Choung Y.-H. (2013). Clinical significance of β-tricalcium phosphate and polyphosphate for mastoid cavity obliteration during middle ear surgery: Human and animal study. Clin. Exp. Otorhinolaryngol..

[58] Pérez-Tanoira R., García-Pedrazuela M., Hyyrynen T., Soininen A., Aarnisalo A., Nieminen M.T., Tiainen V.-M., Konttinen Y.T., Kinnari T.J. (2015). Effect of S53P4 bone substitute on staphylococcal adhesion and biofilm formation on other implant materials in normal and hypoxic conditions. J. Mater. Sci. Mater. Med..

[59] Timmermans A.J., Quak J.J., Hagen P.J., Colnot D.R. (2019). 18F-NaF PET/CT imaging of bone formation induced by bioactive glass S53P4 after mastoid obliteration. Eur. J. Hybrid Imaging.

[60] Kanemaru S., Umeda H., Yamashita M., Hiraumi H., Hirano S., Nakamura T., Ito J. (2013). Improvement of eustachian tube function by tissue-engineered regeneration of mastoid air cells. Laryngoscope.

[61] Quaranta N., Iannuzzi L., Petrone P., D’Elia A., Quaranta A. (2014). Quality of life after cholesteatoma surgery: Intact-canal wall tympanoplasty versus canal wall-down tympanoplasty with mastoid obliteration. Ann. Otol. Rhinol. Laryngol..

[62] Pontillo V., Ciprelli S., Grillo R., Quaranta N. (2023). Quality of life after revision mastoidectomy with mastoid obliteration. Acta Otorrinolaringol. Esp..

[63] Pontillo V., Damiani M., Harib A., Sammali M., Graziano G., Quaranta N. (2022). Quality of life after cholesteatoma surgery: Comparison between surgical techniques. Acta Otorhinolaryngol. Ital..

